# Recent Analytical Approaches for the Study of Bioavailability and Metabolism of Bioactive Phenolic Compounds

**DOI:** 10.3390/molecules27030777

**Published:** 2022-01-25

**Authors:** Álvaro Fernández-Ochoa, María de la Luz Cádiz-Gurrea, Patricia Fernández-Moreno, Alejandro Rojas-García, David Arráez-Román, Antonio Segura-Carretero

**Affiliations:** 1 Max Delbrück Center for Molecular Medicine in the Helmholtz Association, 13125 Berlin, Germany; 2 Berlin Institute of Health, Metabolomics Platform, 10178 Berlin, Germany; 3 Department of Analytical Chemistry, Faculty of Sciences, University of Granada, Fuentenueva s/n, E-18071 Granada, Spain; mluzcadiz@ugr.es (M.d.l.L.C.-G.); patrifdez@correo.ugr.es (P.F.-M.); alejorogar@ugr.es (A.R.-G.); ansegura@ugr.es (A.S.-C.)

**Keywords:** analytical chemistry, bioactive compounds, bioavailability, metabolism, phytochemicals, phenolic compounds, metabolomics, chromatography, mass spectrometry, untargeted

## Abstract

The study of the bioavailability of bioactive compounds is a fundamental step for the development of applications based on them, such as nutraceuticals, functional foods or cosmeceuticals. It is well-known that these compounds can undergo metabolic reactions before reaching therapeutic targets, which may also affect their bioactivity and possible applications. All recent studies that have focused on bioavailability and metabolism of phenolic and terpenoid compounds have been developed because of the advances in analytical chemistry and metabolomics approaches. The purpose of this review is to show the role of analytical chemistry and metabolomics in this field of knowledge. In this context, the different steps of the analytical chemistry workflow (design study, sample treatment, analytical techniques and data processing) applied in bioavailability and metabolism in vivo studies are detailed, as well as the most relevant results obtained from them.

## 1. Introduction

Bioactive compounds are characterized by exerting a biological activity that leads to metabolic alterations associated with beneficial effects on human health, such as the improvement of certain physiological functions or the reduction of the risk of suffering from various diseases. Plant sources generally contain a high content of bioactive compounds, with a wide variety of substances with different chemical structures and biological activities. Some examples of these compounds that present benefits in human health are minerals; vitamins; and other non-nutrient compounds called phytochemicals, such as folic acid, carotenoids, terpenoids, phenolic compounds, glucosinolates or phytosterols, among others [1,2].

Due to advances in the knowledge of bioactive compounds, new products are being developed, such as functional foods, nutraceuticals or cosmeceuticals, with the aim that the consumption contributes beneficial effects to health, in addition to allowing a revaluation of possible agri-food by-products [3,4]. Traditionally, a bottom-up methodology has been carried out to assess the bioactivity of the potential extracts or individual compounds present in vegetal sources. In brief, in this traditional approach, in vitro assays using different enzymatic or cell models have usually been the starting point in order to evaluate the bioactivity of specific extracts or compounds from plant sources [5,6]. Generally, these studies are applied as a screening tool to select the most bioactive extracts or compounds to continue studying those using in vivo models. Many of these studies have demonstrated the pleiotropic character of many bioactive compounds, which are capable of presenting beneficial effects on various therapeutic targets [7]. On the other hand, there are also in vitro studies that have attributed the bioactivity to a synergistic effect between several compounds; however, the mechanisms of action of these effects should be explored in greater depth [8,9]. Once the potential extracts are also chemically well characterized, these or the compounds that have shown beneficial effects are usually selected for further evaluation in animal models [10,11] and, finally, in clinical trials with humans [12,13].

It is well-known that different bioactive compounds can undergo important transformations during their metabolization, before reaching the target tissues. For example, plant-derived phenolic compounds can undergo diverse digestive transformations driven by microbiota and digestive enzymes to become bioactive [14]. These compounds may be first hydrolyzed by gastric fluids in the stomach and later metabolized by the enzymes of intestinal cells or catabolized by the colonic microflora, which may drastically affect the absorption of these molecules through the gut barrier. They usually get their sugar tails removed, generating aglycons. Dietary phenolic compounds are often conjugated or modified by β-glucosidases, UDP-glucuronosyltransferase or catechol-O-methyltransferase in the small intestine; pass through the portal vein towards the liver; and are modified by a number of phase I and II enzymes in the liver, where they undergo methylation, sulfation or glucuronidation. Therefore, the unaltered compounds could hardly be also considered responsible for the observed effects [15].

Although these metabolization reactions have been known for years, there has been an increase in nutritional intervention studies in recent years, with the aim of achieving a better understanding the bioavailability and metabolism of extracts or bioactive compounds. This increased interest in this field of research may be related to advances in instrumental techniques (i.e., mass spectrometry, nuclear magnetic resonance and chromatographic techniques) [16], data-processing tools and databases [17]. In fact, all of these aspects are integrated in the area of metabolomics, which focuses on the study of low-molecular-weight molecules and has experienced an exponential increase in studies in the last decade [13]. The objective of this review is to describe the latest advances in bioavailability and metabolism studies of bioactive phenolic compounds and terpenoids. Specifically, the review focuses on describing both the methodological aspects and the results that are being obtained through these studies.

## 2. Experimental Designs

Different experimental models have been used to explore the bioavailability, pharmacokinetics and the metabolism of bioactive compounds. In vitro models have been widely used for this purpose. Especially the human cell line Caco-2, which is an intestinal epithelial cell model derived from a colon carcinoma, has been used as an approximation to know the absorption through the intestinal barrier [18]. Numerous studies have used this in vitro model to predict the bioavailability of several terpenoids [19,20] and different families of phenolic compounds, such as flavonoids [6,21], procyanidins [22] or hydroxycinnamic acids [23]. The different applications and current trends based on the Caco-2 cells models have recently been reviewed by Ding et al. [24]. Gastrointestinal digestion models have also been widely used to assess the digestibility, bioaccessibility, pharmacokinetics and metabolism of bioactive compounds. There are different models (e.g., static, dynamic and colonic models) that are mainly based on a series of reactors that simulate the reactions that occur in the gastrointestinal tract from the oral cavity to the colon. Therefore, these models allow us to simulate possible transformations of the original bioactive compounds in their derived metabolites generated during physiological processes [25,26]. Wojtunik-Kulesza et al. recently reviewed the main characteristics of these in vitro digestion models, as well as the main studies carried out for the study of food polyphenols [26]. Considering the large number of studies in this topic, this review is mainly focused on describing the latest advances in in vivo models and clinical trials.

Regarding in vivo assays, human and animal models have been used to evaluate the bioavailability and metabolism of bioactive ingredients. Each of these models has its own advantages and disadvantages. One of the aspects in common with both types of studies is that the ethical aspects established in the corresponding regulations must be met. The ethical recommendations of the Declaration Helsinki are generally taken into account in this type of study, in addition to the possible ethical requirements of each institution or country [27]. Therefore, an approval by an ethics committee is required before the start of the study. Some of these studies have been also registered online in the ClinicalTrials.gov database [28,29]. All of these ethical considerations are important to consider in advance when planning the experiment, since it normally takes time until approval by the ethics committee. Particularly in human models, an informed consent from the volunteers is also required.

Animal models have important advantages depending on the hypothesis of the study. Firstly, there is a greater possibility of analyzing more types of biological tissues and organs (e.g., liver, kidney, brain, etc.), and this is quite relevant for the case of knowing in which organs the compounds are accumulated and can exert their bioactive action. For example, Navarro-González et al. collected liver samples to study the hypocholesterolemic effect of a tomato juice, based on previous studies suggesting this effect due to the inhibitory action of HMG-CoA reductase (HMGCR) [30]. Yuan et al. developed and applied a method to determine the bioavailability of raspberry ketone and its derived metabolites in plasma and brain of mice [31,32]. Despite the possibility of collecting different tissues and organs in animal models, they can normally only be collected once the animals are euthanized. For this reason, plasma, urine and stool samples are generally those collected for the study of bioavailability and metabolism in most animal studies.

Nevertheless, there are also studies that have a different main objective (e.g., evaluate the impact of the bioactive ingredient on metabolism during a prolonged intake, etc.) that collect this type of tissue or organs at the end of the trial and are capable of detecting bioavailable compounds. For example, liver samples were also collected in diabetic rats to evaluate the antioxidants effects of a food supplement based on Mango [33]. In this study, the objective was to evaluate the changes in metabolism in liver and plasma after the consumption of mango extract for one month. Although the objective was not precisely to evaluate the bioavailability, as the liver was collected at the end of the assay, some metabolites from the extract were detected as bioavailable, such as the euxanthone metabolite. This aspect of collecting only samples at the end of the assay to assess bioavailability has been carried out by other studies, especially in animal models [34].

In addition to the different types of biological samples, animal models present greater versatility in terms of experimental designs, such as intestinal perfusion studies. Specifically, these perfusion studies consist of the creation of a small isolated intestinal compartment with the help of syringes and valves in order to introduce the matrix to be studied directly into the intestine, and then collect samples of intestinal content at different times. This in situ experimental design allows the study of the absorption and metabolism of the compounds of interest in the intestinal region [10]. This type of model has been used to study specific phenolic compounds (e.g., oleacein and its derived metabolites [35], oleocanthal [36], etc.) or bioactive extracts (e.g., rosemary [37], mahonia bealei [38], ginger extract [39], etc.).

Moreover, animal studies allow researchers to control many variables, such as diet, age, activity, hydration status, etc. These metavariables can affect the results, especially if the samples are to be analyzed by using an untargeted approach, where the possible signals of interest are not previously known. However, these types of studies in animal models are based on those that evaluate drugs, where some studies have shown a poor correlation between the bioavailability results between the animal model and the human model [40].

It is important to note that a possible application for the revaluation of agro-industrial by-products is for the production of higher quality feed [41,42]. If these feeds are developed for a specific animal species, a bioavailability study should be performed on that species to have a better understanding of the responsible/bioavailable metabolites and their biological mechanisms of action. Although there are dietary intervention studies performed in ruminants that evaluate different effects of the intake [41], no specific bioavailability studies have been found published yet.

Human trials have the main advantage that the results are as similar to those of a future consumer application. However, there are many factors that may be out of control relative to that of a real application. For example, Gómez-Juaristi et al. criticized that previous bioavailability studies had been carried out by using doses that were not realistic to those of a possible application [43]. Therefore, the authors in this study used a more realistic dose by following the recommendations of a cocoa manufacturer. There are also studies that evaluate different doses of the investigated extract. For instance, Rodriguez-Mateos et al. used three different doses of a wild blueberry extract in their acute intervention. Their results showed that the absorption and metabolism of the studied phenolic compounds were not exclusively intake-dependent, thus revealing a complex metabolic fate of these compounds [44].

In order to have greater control over all the variables that may affect the results, inclusion and exclusion criteria are previously defined for the recruitment of volunteers. These criteria are usually based on body mass index (BMI), age range, presence of some pathologies, smoking status, medical history or lifestyle, among other factors. There are also studies that select subjects of only one gender or a very specific population based on the hypothesis of the study. For example, Mueller et al. established a two-group experimental design based on groups of women with and without Crohn’s disease, respectively [45].

One of the aspects that should be controlled the most in a human trial is the diet in the days before and during the intervention. A series of recommendations is usually given to volunteers, with the aim of avoiding products that contain compounds similar to those of the objective of the intervention study during the previous days. In fact, there are studies that describe all of these recommendations in the experimental section [43]. Some studies also detail different recommendations for the three days before and for the day before the study [28]. It is also common to be explicit in the exclusion criteria, establishing aspects related to diet. For example, Shön et al., whose study aimed to determine the bioavailability of compounds from a maqui berry extract, established the high consumption of coffee (>3 cups/day), fruits or vegetables (>5 servings/day) or related dietary supplements (based on vitamin C, vitamin E, proanthocyanidins, etc.) as exclusion criteria for the selection of volunteers [28]. In addition, a form of what they have consumed during the last 24 h at the beginning of the study is also often used for greater control over the diet of the volunteers [43,46]. Apart from that, it is generally required that volunteers come under fasting conditions before starting the dietary intervention [28]. In addition, since the studies take several hours, the additional food and beverages ingested during the intervention have to be closely controlled in order to avoid possible interferences in the results. For example, Mueller et al. detailed all the meals that the participants consumed during their intervention study. They also reflected that various types of compounds, such as sugars or proteins, could interfere with the stability of the compounds under study, which were anthocyanins [45]. Wash-out periods need to be properly defined in studies conducting the intervention of different products or compounds using the same group of volunteers. For example, a 2-week wash out period was defined by Motilva et al. between two dietary interventions [47].

This aspect of the influence of diet prior to and during the study can also be controlled by using a control group. Since these studies do not usually recruit large numbers of volunteers, the sample at time 0 is usually used as a control sample for comparison, which is quite useful in targeted studies that are focused only on the evaluation the previously known bioactive compounds. However, in non-targeted studies that pursue the search for new derived metabolites or changes in the endogenous metabolome, the sample at time 0 may not be enough, since signals can be detected from the diet consumed during the time of the intervention. Therefore, the use of a control group taking a placebo product has been used in several of these studies [48,49,50]. In this regard, both single-blind and double-blind experimental designs have been used in bioavailability and metabolism studies [51,52].

## 3. Biological Samples

As mentioned before, plasma, urine and stool samples are the most commonly used in this type of studies, although other biological fluids (e.g., saliva) or tissues have also been collected and analyzed [31,32,53]. There are many aspects related to the collection and treatment of these types of samples that are general to other metabolomics studies. For example, the conservation of biological samples at a low temperature, generally at −80 °C, until their analysis; the stage of protein precipitation of blood plasma/serum samples; centrifugation of urine samples; and others. Many of these conditions have been reflected in previous reviews [54,55]. This section focuses on discussing the aspects of collecting these samples in relation to bioavailability and metabolism studies.

**Figure 1:**
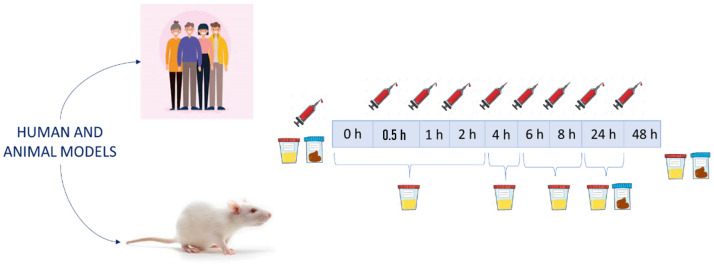
Typical experimental design for the evaluation of bioavailability and metabolism in plasma, urine and stool samples.

Plasma or serum samples are the main ones to reveal that compounds reach the bloodstream by crossing the intestinal barrier and establish the pharmacokinetics curves. The time intervals in which the samples are collected are one of the main aspects that must be carefully designed. Although there are differences between the studies, the most frequent times for collecting blood samples are at 0, 0.5, 1, 2, 4, 6, 8 and 24 h (Figure 1). Some of the less recent studies used sample collection times that did not reach 8 h after the intake [44,56]. Some of these studies showed the importance of catabolism in the colon. In particular, Pimpao et al. detected the presence of simple phenols that could be absorbed from the colon and reach the bloodstream [56]. Mueller et al. used two experimental groups (with and without colon) to investigate the role of the colon in the absorption of phenolic compounds. Their results indicated that the colon plays a significant role for the absorption of anthocyanins and their derivate metabolites [45]. Due to these results, the role of the microbiota in the metabolism and absorption of phenolic compounds is currently well-known. For this reason, plasma samples are usually collected up to 8 h after the intervention in most studies or even longer [46,57,58]. For example, Mecha et al. explicitly mentioned that the metabolites detected after 8 h refer to colonic bacterial metabolism [58].

Urine samples are of special interest, since the excreted metabolites may be a reflection of the metabolic transformations produced in the original compounds of the extract. Regarding this type of sample, there are studies that use only a single interval of sample collection [59,60] and studies that use multiple collection intervals [58,61]. In general, urine samples are usually collected after establishing several time intervals for better monitoring of metabolite excretion. In the case of urine in human models, it is mostly collected at the following intervals: (0–2), (2–5), (5–8) and (8–24) h. In animal assays, they are usually collected at the end of the experiment. Similar to plasma samples, some authors recommend establishing the urine sample collection up to 48 h after ingestion in order to have a greater coverage of all possible microbial metabolites [43].

One of the main aspects to consider in urine samples is that the volume excreted depends on the hydration status of the individuals. To correct this effect, different normalization methods have been described in the literature [62]. This aspect is particularly challenging for spot aliquots of urine. However, in most bioavailability studies, all excreted urine is collected during the established study time or during specific intervals. This fact is a great advantage, since the samples can be easily normalized according to the volume excreted. In this regard, Mecha et al. measured the excreted volumes of each interval and then added L-ascorbic acid as an internal standard to achieve a concentration of 0.5 g/L in each aliquot [58]. Wiese et al. measured the creatinine level in the urine samples because this metabolite has widely used as a marker for normalization in samples from healthy volunteers [63].

Lyophilization techniques have been used in some studies to treat urine samples [47]. This method is generally used for the treatment of feces samples in order to homogenate them [63]. Although the compounds present in stool samples have not been absorbed by the intestinal tract, they may reflect reaction products of the colonic microbial metabolism [64]. Understanding the interactions between bioactive food compounds and gut microbiota is essential to know in more detail about bioavailability and, above all, the biological functions and health benefits of these bioactive molecules [65]. It has been described that diet and, more especially, bioactive compounds play a fundamental and reciprocal role in modulating the microbiota. Therefore, it is very important to know which metabolites are those that have interacted with it when crossing the intestinal colon [66]. Furthermore, due to the role described by the microbiota in the modulation of different diseases [67], this information is increasingly relevant, as well as the studies that correlate these results with microbiomics studies [68].

Some studies have used extraction techniques, such as solid-phase extraction (SPE), for phenolic compounds extractions from the biological samples [47,59]. This technique has the advantages of increasing analytical selectivity and reducing possible interferences and matrix effects. However, depending on the type of cartridge, all the metabolites of interest may not be properly extracted. For example, Luis Ordoñez et al. concluded that styrene divinylbenzene (SDB-L) and hydrophilic/lipophilic-balanced (HLB) cartridges are not suitable for sulfated derivatives, due to low recoveries [59]. In contrast, good results were obtained for free phenolic compounds, as well as for glucuronized metabolites.

It is also important to note that enzymatic cleavage of glucuronides and sulfates using β-glucuronidase and sulfatase have been employed in some studies [44,69]. The main reason of this reactions is to detect and quantify the total amount of each compound (aglycones plus derived metabolites) [63]. Rodriguez-Mateos et al. treated the plasma samples based on an enzymatic treatment with β-glucuronidase and sulfatase, which was reported as a limitation, since the information related to the derived metabolites was lacking [44]. These enzymes have been also used in other studies for the analysis of phenolic compounds in urine or feces samples [63,70]. However, considering the previous appreciation, its use is not recommended, due to the loss of information regarding the derived metabolites.

## 4. Instrumental Techniques

Mass spectrometry (MS) and nuclear magnetic resonance (NMR) are the most used techniques in metabolomics studies. Furthermore, chromatographic techniques, especially liquid chromatography (LC) or gas chromatography (GC), are of great importance, especially in metabolomics, when they are used coupled to MS, since they are capable of producing a previous separation of the compounds, improving selectivity and reducing complexity and matrix effects [71]. All of these techniques also have a fundamental role in different stages for the development of functional foods [16]. Despite the versatility of analytical techniques, most bioavailability and metabolism studies have mainly been based on high- or ultrahigh-performance liquid chromatography coupled with mass spectrometry (HPLC–MS and UHPLC–MS, respectively). Given the great advantages of LC–MS detection, the diode array detector (DAD) is less and less used in this type of study. In fact, some studies have used it but also with a coupling to an MS detector (LC–DAD–MS) [28,63]. Some studies have used this DAD detector to quantify some specific phenolic compounds [63]; on the other hand, there are also studies that have used it to monitor possible contaminations and for general quality assurance purposes [28].

Because of the lower sensitivity of NMR compared to MS, it is not generally selected for this bioavailability and metabolism studies. Therefore, most studies are inclined to use MS for the detection of bioavailable metabolites in biological samples, since it is possible to detect them at lower concentrations in biological samples [72]. However, NMR has a fundamental role for the structural elucidation of metabolites. For example, this technique has been used in studies that synthesize some of the metabolites to confirm their identities [73].

Furthermore, it is notable that GC–MS is not generally used in this type of study. Luis-Ordóñez et al. evaluated GC–MS and HPLC–MS techniques for the analysis of microbial metabolites in urine [59]. They concluded that GC–MS is not a suitable technique for the analysis of phase-II-derived metabolites, such as sulfated or glucuronized phenolic metabolites. This fact was reported because of the lack of volatility of these compounds, even after being derivatized with N-Methyl-N-(trimethylsilyl)trifluoroacetamide (MSTFA). They verified that the glucuronides and sulfated metabolites were recovered intact after the derivatization reaction. Despite this limitation, there are reported studies that have used this instrumental technique to study the metabolism and bioavailability of phenolic compounds [63,74]. These studies are primarily based on targeted studies focusing on analyzing free phenolic compounds and microbial-derived phenolic catabolites, such as hydroxycinnamic and hydroxybenzoic acids and their derivatives [75]. Given the relative polarity of these metabolites, GC–MS is considered a suitable technique for their analysis. Due to the limitation of analyzing phase-II-derived metabolites, the use of enzymatic reactions to deconjugate phase-II derivatives has also been used as part of the sample treatment for determinations based on GC–MS [74]. In spite of these enzymatic reactions, this strategy provides little information about the metabolization reactions of phenolic compounds.

Considering the limitations of NMR and GC–MS mentioned above, we can see that LC–MS has been the most widely used analytical platform in bioavailability and metabolism studies of phenolic compounds. Regarding the LC technique, HPLC and UHPLC instruments have been used in these studies [76]. UHPLC is characterized by resisting higher pressures, thus allowing the use of columns with smaller particle size that achieve higher chromatographic resolution and shorter analysis times, among other advantages [77]. Regarding the working mode, the reversed-phase (RP) mode using a C18 column is the most used in this type of study, since this type of column has been shown to be a good option for the analysis of multiple phytoconstituents, such as phenolic compounds and their derivatives [78].

Regarding MS conditions, most studies have used an electrospray ionization source, generally in negative mode, since this is the most appropriate ionization mode for most phenolic compounds [79]. Nevertheless, some studies have also used the positive ionization mode for the detection of specific compounds [80,81]. For example, Iglesias-Carres et al. used the positive mode for anthocyanidins metabolites detection [80].

There are differences in the MS analyzers between the published studies. These differences are generally related to the type of metabolomic approach selected. On the one hand, some studies have been based on targeted methods focusing only on previously known compounds, such as the free phenolic compounds characterized in the extracts or possible known derived metabolites [28,31,32]. For example, Yuan et al. developed and used a targeted method for the analysis in mouse plasma and brain of raspberry ketone and 25 derived metabolites [31,32]. Since these metabolites were previously known, low-resolution MS analyzers have been used for their identification and quantification in biological samples. The triple quadrupole MS analyzer (QQQ) and quadrupole ion trap (Q-IT) are the most widely used [47,73,82]. These tandem mass analyzers (MS/MS) allow for the selection of specific transitions of the metabolites of interest. The modes are based on selective reaction monitoring (SRM) or multiple reaction monitoring (MRM) which provide high selectivity and sensitivity [61,83]. For instance, Castello et al. optimized a method in SRM mode based on 160 compounds related to the metabolism of anthocyanins, flavonols and phenolic acids. Nevertheless, there are also targeted studies that have used high-resolution MS analyzers, such as time of flight (TOF) or Orbitrap [60,84]. For example, Pereira-Caro et al. developed and validated a UHPLC–ESI-Q-Orbitrap-MS method for the identification and quantification of flavan-3-ol metabolites in biological samples after the intake of a red wine extract rich in proanthocyanidins [60].

On the other hand, untargeted methods pursue a global analysis of all possible signals, without knowing their identity in advance. This approach has the advantage of discovering novel metabolites [49]. For this purpose of identifying new metabolites, it is necessary to use high-resolution MS (HRMS) analyzers, such as time of flight (TOF) or Orbitrap. These high-resolution analyzers are capable of providing very accurate mass values, as well as the isotopic distributions of the detected ions. All of this information, together with the fragmentation spectra, is very useful for the prediction of the molecular formula and later the identification or annotation of new metabolites [85]. These data are usually compared to those registered in databases, such as human metabolome database (HMDB), kyoto encyclopedia of genes and genomes (KEGG), METLIN, LIGAND, FooDB or massbanks [48,84].

In bioavailability and metabolism studies, there is usually a certain knowledge of the ingested compounds, as well as possible potential metabolites. Therefore, non-targeted studies per se are not generally applied for these objectives. Instead, semi-targeted studies, which combine different characteristics of the targeted and non-targeted methods, are more frequently carried out for the study of bioavailability and metabolism. These studies are based on the analysis of previously well-known compounds and of possible hypothetical derived metabolites that are not discovered prior to the study [43,48,86].

## 5. Data Processing

Typical pharmacokinetic parameters, such as maximum plasma concentration (C_max_), area under the plasma concentration–time curve (AUC), time to reach Cmax (t_max_) or half-life of elimination (t_1/2_), are generally calculated in bioavailability and pharmacokinetics studies [76,87]. Some specific software programs or tools have been used to calculate these parameters, such as the PKSolver tool from Microsoft Excel [88]. These parameters are quantitative in nature, and their calculation requires the concentration values of the metabolites determined in the biological samples through analytical techniques. For this reason, targeted methods focused on quantifying metabolites are of great relevance in this type of studies. This quantification is usually carried out through calibration curves, using standards when available [87]. When these are not available, they are either chemically synthesized [89] or generally determined by using a standard with a similarity in chemical structure [87]. However, this last approach does not guarantee accurate quantitative values, since it is a tentative quantification.

In quantitative targeted methodologies, the validation of the analytical method is a fundamental aspect to guarantee that the concentration values obtained are precise and accurate. In this regard, different bioanalytical-method validation guidelines have been followed in several bioavailability studies [31,60,90]. The main guidelines followed in this type of study have been the Guidelines from the European Medicines Agency (EMA), the Eurachem Guide and the US Food and Drug Administration (FDA) [91,92]. For instance, Kundisová et al. validated the following parameters according to the EMA guideline: limit of quantification (LOQ), linearity, matrix effect, recovery, selectivity, carry over, precision and accuracy [90]. In another study, Pereira-Caro et al. developed and validated a method for the analysis of flavan-3-ol metabolites in different biological samples. They validated the following parameters according to Eurachem guideline: matrix effects, precision, recovery, linearity, specificity and the limits of detection and quantification [60]. The limits of identification (LOD) and quantification (LOQ) are highly relevant parameters, since they refer to the analytical sensitivity of the method. These are usually calculated through an analysis of blank samples, although there are different estimation criteria based on the different validation guidelines. For example, the LOQ has been calculated differently in bioavailability studies, considering five times the signal detected in a blank sample [90] or 10 times the signal-to-noise ratio (S/N) [60]. In general, current validated targeted analytical methods based on MRM transitions are obtaining LOQ values of up to 0.04 nM [90]. These low values indicate the great capacity of current analytical techniques to detect very low concentrations in biological samples. It is important to mention that the LOQ is a specific parameter of each metabolite analyzed in a specific biological matrix, and, in fact, some studies have obtained a quite variable range of LOQ between the metabolites detected. For example, Pereira-Caro et al., who validated an analytical method for 27 compounds, obtained the following wide LOQ ranges for plasma, urine and feces matrices, respectively: 3–1540, 3–2160 and 3–3110 nM [60]. Due to the importance of the analytical method validation to ensure the quality of the results, some studies have separately published the validation of the analytical method. For example, Yuan et al. developed and validated a targeted method to determine raspberry ketone metabolites, following the FDA guidelines [31]. They validated the matrix effects, recovery, processing efficiency, sensitivity, dynamic range, repeatability and precision. Once the method was validated, it was successfully applied for the bioavailability study of raspberry ketones and their metabolites in mice of different gender [32]. They specifically studied the influence of diet-induced obesity on the bioavailability of the validated metabolites.

Regarding the non-targeted approaches, some studies have followed the identification of metabolites from the prediction of potential metabolites according to the most frequent phase I and II metabolization reactions. In contrast, other studies have followed an untargeted data-processing workflow, based on the stages of peak picking, noise reduction, signal filtering, alignment of retention times and *m*/*z* values, normalization, gap-filling, etc. [48,84]. To carry out this untargeted methodology, there are different software, programs, both commercial and open access, such as mzMine [93] and R or python packages [94]. One of the main aspects of non-targeted studies is the metabolite identification/annotation stage, as mentioned in the previous section. Since this identification stage still presents certain limitations within the entire untargeted workflows, new approaches are being used for the identification of natural products. MS-based molecular networking is one of these novel approaches [95]. This method is focused on the creation of networks based on the similarity of the MS spectra between compounds. The creation of these networks presents advantages for the interpretation, visualization and identification of new metabolites. Although this type of methodology has not yet been widely used in bioavailability studies, Hakeem Sais et al. used this molecular networking approach to identify phenolic compounds’ metabolites from cocoa in urine samples [49].

There is also a variety of statistical tests used in these studies, with the most frequent being univariate analyses, such as analysis of variance (ANOVA) and non-parametric Wilcoxon test [43,76]. Recently, multivariate statistical methods, such as principal component analysis (PCA), partial-least-squares discriminant analysis (PLS-DA), hierarchical clustering analysis or heatmaps, have been also being applied to identify biomarkers from ingestion, especially in untargeted screening studies [96].

## 6. Recent Advances and Future Trends

Although many relevant methodological aspects were discussed in the previous sections, Table 1 and Table 2 summarize the main studies carried out in animal and human models, respectively, in recent years. These manuscripts were selected based on a bibliographic search in PubMed/MEDLINE and Scopus. This literature search was performed by using the terms “Bioactive compounds” or “Phenolic compounds” or “Polyphenols” and “animal” or “human” or “in vivo” or “clinical trial” and “bioavailability”, “metabolism”, “pharmacokinetics”, “bioavailable”, “absorption”, “metabolites”, “glucuronides”, “sulfated”, and “methylated” or “microbial”. Scientific articles from the last 5 years were prioritized and manually supervised for inclusion in the discussion of this review.

**Figure 2:**
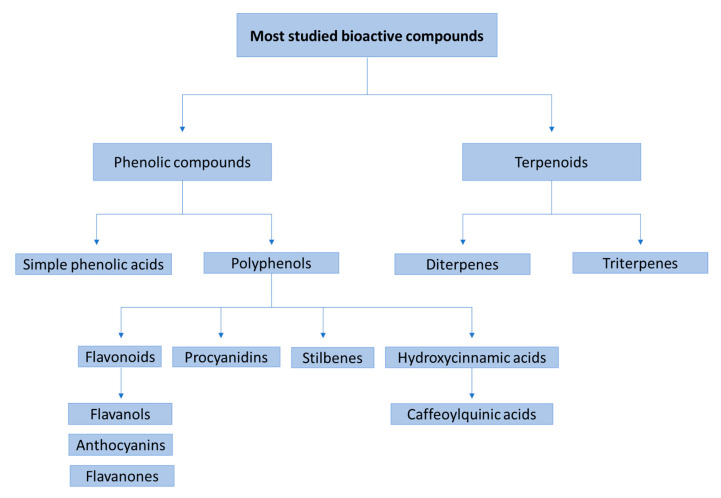
Main families of phenolic compounds that have been studied through bioavailability and metabolism studies.

In general, the main studied families of bioactive compounds are terpenoids and phenolic compounds, including simple phenolic acids and polyphenols, such us flavonoids (flavanols, anthocyanins and flavanones), procyanidins, stilbenes and hydroxycinnamic acids (e.g., caffeoylquinic acids) (Figure 2). There is a wide variety of different matrices studied in recent studies. For example, there are studies that focus on the evaluation of the bioavailability of individual compounds [83], bioactive extracts [37], beverages [30,79], foods [58] or food supplements [63]. The most common studied extracts come from orange juice, cocoa, grape pomace and berry fruits, among others (Table 1 and Table 2). After ingestion, the bioactive compounds and their metabolites were assessed mostly by using UHPLC– and HPLC–ESI–MS/MS techniques.

**Figure 3:**
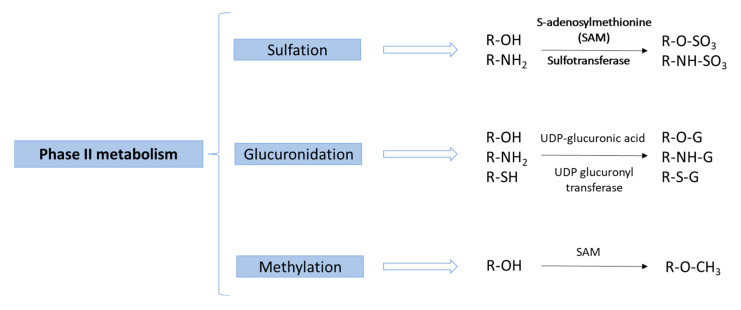
Main phase II metabolism reactions of phenolic compounds.

In these studies, according to what was expected in relation to the previous knowledge about the metabolism of phenolic compounds [97], most of the investigated compounds undergo phase II metabolism, especially through sulfation, glucuronidation and methylation reactions (Figure 3). Because of all the methodological progress mentioned on this type of study, new metabolites derived from phase I or phase II metabolism have recently been identified in biological samples (see Table 1 and Table 2). For example, Achour et al. showed that most rosemary phenolic compounds were metabolized into phase II metabolites, which were detected in human biological samples. In contrast, only a few original compounds were detected as being bioavailable [86]. In this regard, many secondary metabolites have been detected for the first time in biological samples [57,70].

**Table 1:** Recent advances in bioavailability and metabolism studies carried out in animal models.

Matrix	Bioactive Compounds	Model (Nº Animals/Volunteers)	Biological Samples	Collection Times	Technique (Column)	Relevant Results (Metabolites, Reactions, etc.)	Reference
Rosemary extract	Flavonoids, diterpenes and triterpenes	Mice model (in situ perfusion assay) (n = 7)	Gastrointestinal liquid	5, 10, 15, 20, 25, 30 min	HPLC–ESI–QTOF-MS (RP-C18)	Several diterpenes and four new metabolites detected in plasma. Sulfation and glucuronidation reactions.	[37]
			Plasma	End of the assay			
Ginsenoside Rb1	Ginsenoside Rb1, Impact of 3 different fibers	Male Sprague Dawley rats (n = 32)	Plasma	0, 0.25, 0.5, 1, 1.5, 2, 3, 4, 6, 8, 10, 12, 24, and 48 h	UHPLC–ESI–QQQ-MS (RP-C18)	Secondary ginsenosides, especially ginsenoside CK, are the major active metabolites. Prebiotics promote the proliferation of certain bacterial strains that improve the biotransformation and bioavailability of ginsenosides.	[76]
			Feces	-14 d, 0 h and 48 h			
Quercetin glucoside mixture supplement	Quercetin glucoside (Quercetin-3-O-glucoside) and its glucose adducts	Male Wistar/ST rats (n = 35)	Plasma	Once in weeks 2, 4, 6, 8	HPLC–ESI–QQQ-MS (RP-C18)	Three phases of quercetin metabolism, including cumulative, transient, and stable phases revealed. Water-soluble dietary fibers, especially soybean fiber, enhanced quercetin bioavailability.	[69]
			Urine	Two times in weeks 2, 4, 6, 8			
			Feces	Three times in weeks 2, 4, 6, 8			
Tomato juice	Lycopene, naringenin and chlorogenic acid	Sprague Dawley rats (n = 16)	Plasma	End of experiment	HPLC–ESI–IT-MS (RP-C18)	Total cholesterol was lower after the intervention. Low bioavailability of chlorogenic acid and naringenin.	[30]
			Urine	Daily for 5 weeks			
			Feces	Daily for 5 weeks			
			Liver	End of experiment			
Arbequina table olives	Hydroxytyrosol, tyrosol, verbascoside, luteolin, salidroside and *p*-coumaric acid	Male Sprague-Dawley rats (n = 7)	Plasma	0, 30 min	HPLC–ESI- QIT-MS (RP-C18)	The possible metabolism suffered in the enterocytes cannot be underestimated. Importance of different mechanisms of absorption depending on the hydrophilic or lipophilic nature of the analyte.	[90]
Red raspberry	Raspberry Ketone (4-(4-hydroxyphenyl)-2-butanone))	Mice (Non-specified)	Plasma	End of experiment	UHPLC–ESI– QQQ-MS (RP-C18)	25 analytes identified as RK-derived metabolites.	[31]
			Brain	End of experiment			
Extra virgin olive oil (EVOO)	Oleocanthal (OLC)	Sprague-Dawley rats (n = 4)	Intestinal fluid	Every 5 min for 60 min	UHPLC–ESI-QQQ-MS (RP-C18)	Metabolism of phase I and II. Higher levels of OLC are expected to reach human plasma vs. rat plasma.	[36]
			Plasma and intestinal lumen	End of experiment			
Red grape polyphenols	Flavanols, phenolic acids, cinnamic acids, valerolactone and valeric acid	Wistar rats (n = 12)	Serum	0, 2, 4, 7, 24, 48 h	HPLC–ESI–QTOF-MS (RP-C18)	Organic cultivation system influences the bioavailability and metabolism of polyphenols. Phase II metabolites.	[80]
Red grape polyphenols	Cinnamic acid, benzoic acid, flavonoid, phenylpropionic and phenylacetic acid	Male Fischer-344 rats (n = 54)	Serum	End of experiment	HPLC–ESI–QTOF-MS (RP-C18)	Flavonoid phase II metabolites. 6 h of light per day improves bioavailability of phenolic compounds.	[81]
Calafate berry extract	Anthocyanins and hydroxycinnamic acids	Gerbils (n = 18)	Plasma	0, 1, 2, 4, 8, 12 h	GC–EI- QQQ-MS (HP-5MS)	ß-oxidation products were detected. Hydroxycinnamic, benzoic, and phenylacetic acids derivatives. No parental anthocyanins were detected.	[74]
Red wine extract.	Flavan-3-ols, proanthocyanidins	Male Sprague-Dawley rats (n = 3)	Plasma	24 h	UHPLC–ESI–Q-Orbitrap-MS (RP-C18)	Phase II metabolism. Importance of the colonic microbiota in the transformation of proanthocyanidins.	[60]
			Urine	24 h			
			Feces	24 h			
Corylin extract supplement	Corylin metabolites	Male SPF grade KM mice (n = 18)	Plasma	0.5, 6 h	UHPLC–ESI–QTOF-MS (RP-C18)	Phase I metabolism of corylin. Oxidation, hydration, glucuronidation and sulfation reactions.	[34]
			Urine	End of experiment			
			Feces	End of experiment			
			Bile	End of experiment			
Grape pomace	Phenolic acids and anthocyanins	Male rats (n = 30)	Urine	0, 6 and 14 months	UHPLC–ESI–QTOF-MS (RP-C18)	Methylated, sulfated and glucuronidated metabolites. Growth inhibition of Clostridium.	[98]
Malaxinic acid and its aglycone	Malaxinic acid (MA) and its aglycone (MAA)	Male Sprague-Dawley rats (n = 50)	Plasma	0, 15, 30, 60, 120, 240, 480 min	HPLC–ESI–Q-IT-MS (RP-C18)	Absence of intact forms of MA and MAA. Glucuronide metabolites were detected.	[73]
Rice bran enzymatic extract	Ferulic acid	Male Wistar rats (n = 50)	Plasma	0, 15, 30, 60 min 3, 6, 12, 18, 24 h	UHPLC–ESI–QQQ-MS (RP-C18)	Sulfated metabolites and unconjugated simple aromatic acids. Phase II metabolites.	[61]
			Urine	0, 1, 2, 3, 4, 5, 6, 9, 24, 48 h			
			Feces	0, 24, 36, 48 h			
Specific phenolic compounds	Hydroxytyrosol, hydroxytyrosol acetate, DOPAC	Sprague-Dawley rats (n = 120)	Plasma	0, 0.5, 1, 2, 4, 8, 24 h	UHPLC–ESI–QQQ-MS (RP-C18)	Influence of the sex-linked metabolism on the excretion pattern. The amounts of bioactive compounds did not result in a proportional increase in their plasma concentrations.	[83]

CK, compound K; DOPAC, dihydroxyphenyl acetic acid; EI, electronic impact; ESI, electrospray ionization; EVOO, extra virgin olive oil; GC, gas chromatography; HPLC, high-performance liquid chromatography; IT, ion trap; KM: Kunming mice; MA, malaxinic acid; MAA, malaxinic acid aglycone; MS, mass spectrometry; OLC, oleocanthal; Q, quadrupole; QQQ, triple quadrupole; QTOF, quadrupole time of flight; RP, reversed phase; RK, raspberry ketone (4-(4-hydroxyphenyl)-2-butanone)); SPF, specific pathogen-free; UHPLC, ultrahigh-performance liquid chromatography.

**Table 2:** Recent advances in bioavailability and metabolism studies carried out in human models.

Matrix	Bioactive Compounds	Model (Nº/Volunteers)	Biological Samples	Collection Times	Technique (Column)	Relevant Results (Metabolites, Reactions, etc.)	Reference
Rosemary tea	Phenolic acids, flavonoids,	Healthy human volunteers (n = 12)	Plasma	0, 0.5, 1, 1.5, 2, 3, 4, 5, 6, 8, 9, 10 h	HPLC–ESI–QTOF-MS(RP-C18)	Phase II metabolites bioavailables. Metabolism by colonic microbiota.	[86]
			Urine	(−2,0), (0–2), (2–5), (5–8), (8–12), (12–24) h			
Two cocoa products	Flavanols	Healthy human volunteers (n = 13)	Plasma	0, 0.5, 1, 2, 3, 4, 6, 8 h	HPLC–ESI–QTOF-MS(RP-C18)	Phase II derivatives of epicatechin, phenyl-valerolactone and phenylvaleric acid. Importance of colonic reactions.	[43]
			Urine	(−2,0), (0–4), (4-8), (8–12), (1–-24) h			
Cocoa products	Phenolics, flavanols	Healthy human volunteers (n = 13)	Urine	0, 6, 9, 12, 24, 30, 36, 48 h	UHPLC–ESI–QTOF-MS (RP-C18)	Use of multivariate analyses (PCA and PLS-DA) to identify bioavailable compounds Phenyl-valerolactone metabolites. Phase II conjugated metabolites.	[96]
Bilberry pomace extract	Anthocyanins	Healthy women and women with Crohn’s disease (n = 10)	Plasma	0, 1, 2, 4, 8 h	HPLC–ESI–QQQ-MS/MS (RP-C18)	Glucuronides and sulfated metabolites were detected in plasma and urine samples. Higher bioavailability in presence of an intact gut, revealing its potential site of action.	[45]
			Urine	(−24–0), (0–2), (2-–4), (4–8), (8-–24) h			
			Ileostomy fluid	(−12–0), (0–1), (1–2), (2–4), (4–6), (6–8) h			
Cranberry juice cocktail	Flavonoids, phenolic acids and proanthocyanidins	Healthy men and postmenopausal women (n = 10)	Plasma	0, 0.25, 0.5, 1, 2, 3, 4, 5, 6, 10 h	HPLC–ESI–QQQ-MS (RP-C18, RP-C12)	Presence of PAC-A2 dimers in urine. Rapid phase II transformation and excretion of anthocyanins.	[70]
			Urine	0, 2, 4, 6, 8, 10, 24 h			
Instant green/ roasted coffee	Hydroxy-cinnamates	Healthy human volunteers (n = 12)	Plasma	0, 0.5, 1, 1.5, 2, 3, 4, 5, 6, 8, 9, 10, 12 h	HPLC–ESI–QTOF-MS (RP-C18)	Sulfate, methyl and glucuronides metabolites were detected. Dihydrohydroxycinnamate esters have been identified for the first time in both plasma and urine.	[57]
			Urine	(−2–0), (0–2), (2–5), (5–8), (8–12), (12–24) h			
Yerba mate infusion	Caffeoylquinic acids, ferulic acids and hydroxyl-cinnamic acids	Healthy human volunteers (n = 12)	Plasma	0, 0.5, 1, 1.5, 2, 3, 4, 5, 6, 8, 9, 10, 12 h	HPLC–ESI–QTOF-MS (RP-C18)	Sulfated conjugates of caffeic and ferulic/isoferulic acids. Phase II flavanol and phenolic acids metabolites.	[46]
			Urine	(−2–0), (0–2), (2–5), (5–8), (8–12), (12–24) h			
Mixed berry fruit pureé	Caffeoylquinic acids and anthocyanins	Healthy human volunteers (n = 13)	Plasma	0, 0.5, 1, 2, 4, 6 h	HPLC-ESI-QQQ-MS/MS (RP-C18)	Presence of methylated, sulfated and some dual conjugated compounds. Importance of catabolism in the colon.	[56]
Beverage enriched with grape pomace extract	Procyanidins, phenolic acids and flavanols	Healthy human volunteers (n = 12)	Urine	0, 24 h	HPLC–ESI–Q-Orbitrap-MS (RP-C18)	Methylation, sulfation, glucuronidation, hydroxylation, dehydrogenation and glycine conjugation reactions. Seventy metabolites identified.	[50]
Red wine enriched with a grape pomace extract	Phenolic acids, flavanols, stilbenes, anthocyanins and phenyl alcohols.	Healthy human volunteers (n = 12)	Plasma	0, 0.5, 1, 2, 4, 6 h	UHPLC–ESI–QQQ-MS (RP-C18)	Intense phase II metabolism. Sulfated form predominated over the glucuronidated one. Novel endogenous production pathway of hydroxytyrosol metabolites.	[47]
			Urine	(0–6), (6–12), (12–24) h			
Orange juice	Flavanones, flavones and phenolic acids	Healthy human volunteers (n = 9)	Plasma	0, 1, 2, 3, 4, 5, 6, 8 h	UHPLC–ESI–QQQ-MS (RP-C18)	Phase II sulfate, glucuronide, and methyl metabolites. Dehydroxylation and demethoxylation mediated by the gut microflora.	[79]
			Urine	(0–2), (2–5), (5–10), (10–15), (15–24) h			
Cocoa rich in polyphenols	Epicatechin, valerolactones and flavonols	Healthy human volunteers (n = 15)	Urine	0, 3, 6, 9, 12, 24, 30, 36, 48 h	UHPLC–ESI–QTOF-MS (RP-C18)	Phase II conjugation into sulfated and glucuronide derivatives. Bacterial metabolism of cocoa major flavanols.	[49]
Cranberry extract	Phenolic acids, anthocyanins	Healthy human volunteers (n = 13)	Urine	Day 1: 0 h Day 7: 1, 2, 4, 6, 8, 10, 12, 24 h	HPLC–ESI–Q-Orbitrap-MS (RP-C18).	Identification of 42 analytes highlighting the detection of six valerolactones/valeric acid derivatives	[48]
Common beans (*Phaseolus vulgaris* L.)	Flavanols, phenolic acids, catechols and pyrogallols.	Healthy human volunteers (n = 7)	Plasma	0, 1, 2, 4, 6, 8 h	UHPLC–ESI–QTOF-MS (RP-C18)	Glucuronidation and sulfation reactions. Colonic bacterial metabolism of the phenolic compounds was detected. Hippuric acids was the most abundant class of metabolites in urine	[58]
			Urine	0, (0–2), (2–4), (4–6), (6–8), (8–24) h			
Orange juice	Phenolic acids	Healthy human volunteers (n = 3)	Urine	0–24 h	GC–MS and HPLC–ESI-Q-Orbitrap-MS (RP-C18)	Free phenolics and glucuronide and sulfate conjugates were detected. GC–MS was not suitable for the analysis of phenolic sulfate and glucuronide metabolites.	[59]
Maqui berry extract	Anthocyanins (>35%) and delphinidins (>25%)	Healthy human volunteers (n = 12)	Plasma	0, 0.5, 1, 1.5, 2, 3, 4, 6, 8 h	UHPLC–DAD–ESI–QQQ-MS/MS (RP-C18)	Extensive and fast first-pass metabolism. Phenolic acids as breakdown products of anthocyanins were observed.	[28]
Brown seaweed extract	Phlorotannin metabolites	Overweight and obese volunteers (n = 80)	Plasma	Weeks 0, 8, 16, 24	UHPLC–ESI–Q-Orbitrap-MS (RP-C18)	Phase II sulfated and glucuronidated metabolites.	[99]
			Urine	24 h			
Red grape pomace	Anthocyanins, flavan-3-ol monomers, procyanidins	Healthy human volunteers (n = 10)	Plasma	0, 8, 16, 24 h	UHPLC–ESI–QQQ-MS (RP-C18)	Glucuronide and sulfate forms. High inter-individual variability (importance of gut microbiota).	[87]
			Urine	(0–3), (3–6), (6–10), (10–24), (24–36), (36–48) h			
Green tea	Phenyl-γ-valerolactones	Healthy human volunteers (n = 16)	Urine	Day 0, day 8	UHPLC–ESI–QQQ-MS (RP-C18)	Large inter-individual variability due to differences in microbiota patterns. Colonic catabolism of (–)-epigallocatechin and (–)-epigallocatechin-3-gallate.	[89]
Wild blueberry drinks	Anthocyanins, proanthocyanidins, flavonols and chlorogenic acids.	Healthy human volunteers (n = 9)	Plasma	0, 1, 2, 4, 6 h	UHPLC–ESI–QTOF-MS (RP-C18)	23 phenolic acid metabolites were quantified in plasma. Interindividual variability was high (age, dose-dependent effects, gender, gut microbiota and genetic polymorphisms).	[44]
Cranberry juice	Proanthocyanidins, anthocyanins, flavonols and phenolic acids	Healthy human volunteers (n = 10)	Plasma	0, 1, 2, 4, 6, 8, 24 h	UHPLC–ESI–QTOF-MS (RP-C18)	Conjugated and non-conjugated phenolic acid derivatives were detected. Sulfated and glucuronidated metabolites. Phase I and phase II metabolism.	[29]
			Urine	(0–8), (8–24) h			
Seed/fruit extract (*fraxinus angustifolia vahl*)	Secoiridoid glucosides	Healthy human volunteers (n = 9)	Plasma	0, 1, 2, 4, 8, 24 h	UHPLC–ESI–QTOF-MS (RP-C18)	Metabolic conversion by esterases, glycosidases, and phase II sulfo- and glucuronosyl transferases to form smaller conjugated derivatives. Metabolism by phase I and (or) microbial enzymes.	[84]
			Urine	0, (0–8), (8–24) h			
Hard gelatine capsule containing phenolic compounds	Flavan-3-ols (epicatechin, procyanidin B1, and polymeric procyanidins)	Healthy human volunteers (n = 7)	Plasma	0, 1, 2, 4, 8, 24, 48 h	GC–EI-QQQ-MS (DB-5MS) HPLC–DAD–ESI-Q-MS (RP-C18)	Glucuronidated, sulfated and methylated (-)-epicatechin and 5-(3′,4′-dihydroxyphenyl)-valerolactone were the dominant metabolites in blood and urine. High importance of the gut microbiota in flavan-3-ol metabolism.	[63]
			Urine	(0–4), (4–8), (8–24) h			
			Feces	(0–24) h			

DAD, diode-array detection; EI, electronic impact; ESI, electrospray ionization; GC, gas chromatography; HPLC, high-performance liquid chromatography; IT, ion trap; MS, mass spectrometry; PCA, principal component analyses; PLS-DA, partial-least-squares discriminant analysis; Q, quadrupole; QQQ, triple quadrupole; QTOF, quadrupole time of flight; RP, reversed phase; UHPLC, ultrahigh performance liquid chromatography.

Despite the fact that the bioavailability of more and more metabolites derived from the original phenolic compounds is known, the role of these metabolites in relation to bioactive properties is still not fully understood. In fact, to a certain degree, it seems to be redundant to study only the bioactive properties of the original compounds if it is unknown which metabolites are the ones that actually reach the site of action. Therefore, there is an important gap in the knowledge regarding the bioactive properties of derived metabolites. It is expected that, in the coming years, new research studies will focus on studying not only the native compounds per se but also the circulating metabolites to appropriately assign the bioactive properties to a structure [100].

Because of the proven low bioavailability of many phenolic compounds, encapsulation techniques have gained interest in recent years to improve bioavailability of these compounds. The purpose of encapsulation is to protect, transport and release the bioactive compounds in the target areas, thus increasing their bioavailability [101]. Due to the increase in studies focused on the encapsulation of phenolic compounds, there are bioavailability studies that have been carried out to test the bioavailability of encapsulated compounds [102]. Furthermore, considering also the numerous factors that affect the bioavailability (e.g., pH, dose, composition, etc.) of a compound, we see that there are many studies focused on investigating several of these factors. For example, Trakooncharoenvit et al. investigated the effect of different water-soluble dietary fibers on the bioavailability of quercetin-3-O-glucoside metabolites [69]. They showed that the intake of these fibers enhanced the bioavailability of the studied metabolites. Mueller et al. showed that the pH of the intestine adversely affects the stability and absorption of anthocyanins [45]. In another study, the effects of distinct photoperiods, which differ between organically and conventional productions, on the bioavailability of red-grape phenolic compounds were investigated [81].

The determination of the importance of colonic reactions has been one of the most important advances in terms of bioavailability and metabolism of phenolic compounds [43,87]. For instance, Wiese et al. highlighted the high importance of the gut microbiota in flavan-3-ol metabolism [63]. Castello et al. showed the presence of dehydroxylation and demthoxylation reactions mediated by the gut microbiota [79]. Brindani et al. showed the colonic catabolism (–)-epigallocatechin and (–)-epigallocatechin-3-gallate [89]. In addition, a large inter-individual variability due to differences in microbiota patterns has been detailed in some of these studies [87,89]. The production of microbial metabolites, as well as the interaction of the compounds with the bacterial microbiota, is important to know for a better understanding of the potentially bioactive effects of phenolic compounds on an individual basis [87]. In addition, all of these aspects are receiving more and more attention due to the importance of the gut–brain axis, which has been shown to play an important role in the development of certain age-related pathologies, neurodegenerative disorders, etc. [103,104].

All the progress regarding the bioavailability and metabolism of phenolic compounds has been due to advanced analytical techniques, and especially MS. However, according to the continuous progress in analytical techniques in terms of improvements in resolution and sensitivity, it is expected that further progress in this area of knowledge will be achieved because of this progress. For example, despite the great advantages of MS coupled with chromatographic techniques, these methods still have limitations for obtaining the complete characterization of metabolites related to the resolution of isomers that present similar retention times and mass/charge (*m*/*z*) ratios [105]. These limitations seem to have been overcome in recent years, due to the additional coupling of ion mobility spectrometry (IMS) [106]. This technique allows for the additional separation of ions based on their size and charge, discriminating species that are neither separated by chromatography nor by MS. The use of this technique in the field of bioavailability will be of great relevance for the identification of possible specific isomers whose specific chemical structure may be decisive for their relationship with biological properties. These advantages have been shown in different characterization studies of phenolic compounds in different matrices [107,108,109,110]. However, this technique has not yet been applied in bioavailability and metabolism studies, and therefore there is a great future prospect of advancement of knowledge in this area in the coming years.

## 7. Conclusions

The bioavailability and metabolism of phenolic compounds is a key point that is necessary to investigate for a better understanding of the biological action mechanisms, which allow the development of better applications based on these compounds. In recent years, there has been an increase in this type of study, which has managed to identify new bioavailable metabolites in different biological matrices. Phase II metabolism, as well as colonic metabolism, is the most prominent in these advances. All of these advances have been possible due to the continuous progress of analytical and metabolomic approaches. The improvement in terms of selectivity and sensitivity of analytical techniques and, in particular, of LC–MS, has been essential for the detection and quantification of a greater number of bioavailable metabolites in biological samples. However, more advances are expected in the coming years in relation to different aspects related to bioavailability, such as better understanding the bioactive role of metabolized derivatives, evaluation of encapsulation techniques or the use of ion mobility spectrometry.

## Data Availability

Not applicable.
